# Invasive Mole Resulting in Uterine Rupture: A Case Report

**DOI:** 10.3389/fsurg.2021.798640

**Published:** 2022-01-28

**Authors:** Anshan Wu, Qiong Zhu, Chang Tan, Long Chen, Yin Tao

**Affiliations:** Department of Gynecology, Zhu Zhou Central Hospital, Zhuzhou, China

**Keywords:** uterine rupture, Invasive mole, GTN, HCG, mole

## Abstract

Uterine surgery is a common predisposing factor for uterine rupture, while an invasive mole that leads to uterine rupture is a rare clinical occurrence. Here, we report a case of a 31-year-old childless woman who underwent abortion after 53 days of pregnancy. She still experienced abdominal pain and scanty vaginal bleeding after the abortion. Her levels of human chorionic gonadotropin (HCG) were high, while ultrasound and MRI results revealed an enlarged uterus and a mass in the myometrium. During preparation for treatment, the gynecologist ruptured the uterus of the patient, leaving her shocked. Eventually the patient's uterus was removed the uterus and pathologically diagnosed as result is the an invasive mole.

## Introduction

An invasive mole refers to a common manifestation of gestational trophoblastic neoplasia (GTN) that originates from hydatidiform. In the uterine myometrium, invasive moles can grow in the uterine muscular wall and cause massive bleeding in the abdominal cavity (1). Previous studies have shown that the main distant metastasis of an invasive mole occurs in the lungs, with 5% of patients manifesting it in the vagina, pelvis, liver, and brain (1–3). Although an invasive mole is sensitive to chemotherapy and is highly curable (4), delayed treatment has been associated with serious complications, such as uterine perforation and hemoperitoneum (5). Here, we report a case of an invasive mole that caused uterine rupture. From the case, we provide relevant management experience and lessons for future management of invasive moles.

## Case Report

**Figure 1:**
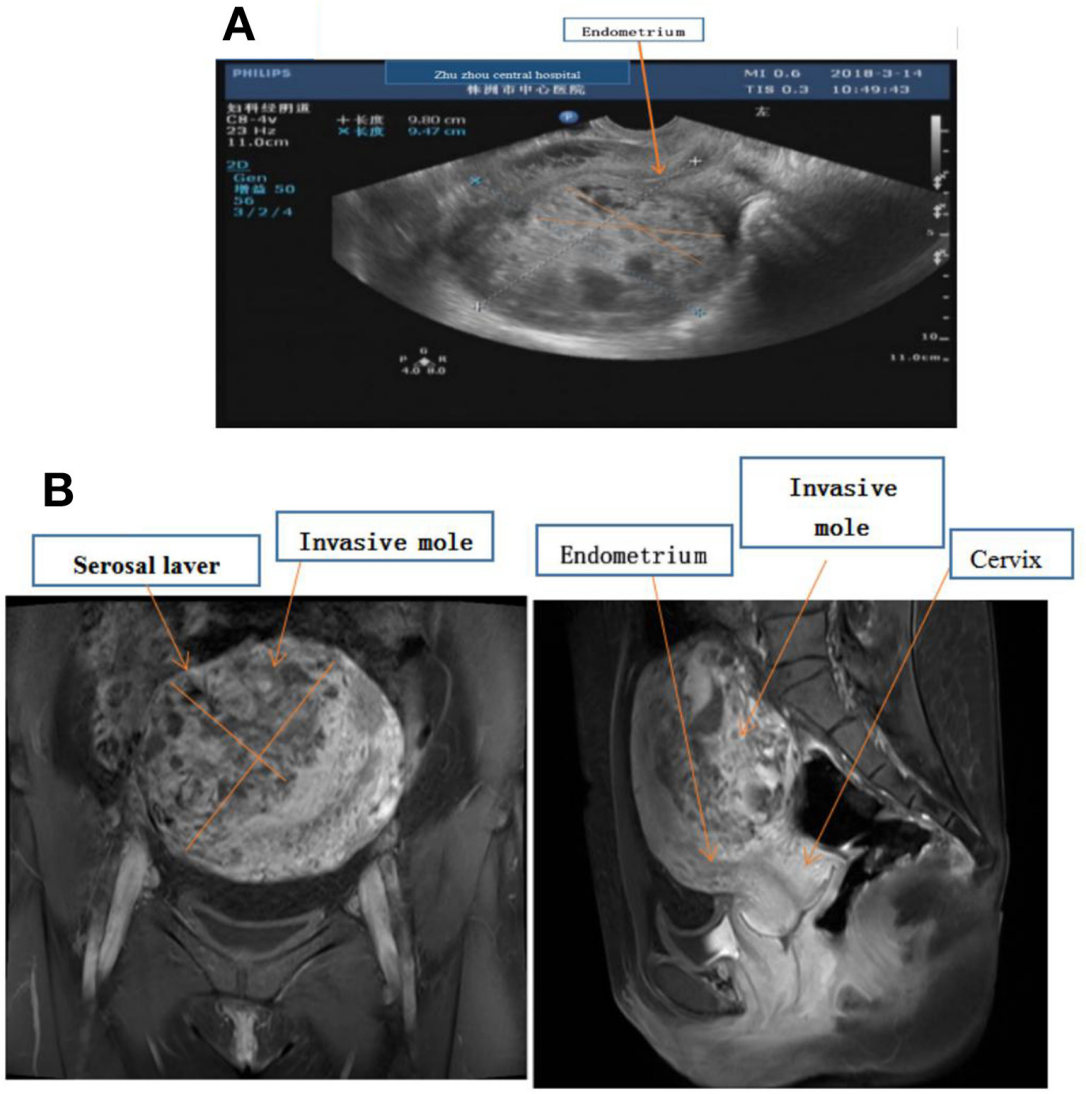
**(A)** Ultrasound examination showing fullness of the uterus, about 98 × 95 × 106 mm, the uterine cavity moved forward, and the endometrial thickness was 4 mm. There was about 85 × 70 × 97 mm hyperechoic masses in the muscular layer of the posterior wall, and the echoes were disordered and uneven. **(B)** MR imaging showing enlarged, uterus 100 × 96 × 105 mm, and there was a mass in the myometrium with the size about 12.3 × 8.0 cm, which invaded the serosal laver but not endometrium.

A 31-year-old woman presented with abdominal pain and scanty vaginal bleeding 53 days post abortion, and was transferred to Zhuzhou Central Hospital. Her serum HCG was 106,189 mIU/ml, while ultrasound examination revealed that her uterus had enlarged to about 98 × 95 × 106 mm^3^, with evidence of a lesion (measuring 12 × 8 cm^2^) in the myometrium (Figure 1A). Pelvic MR imaging also confirmed uterus enlargement to 100 × 96 × 105 mm^3^, and the lesion in myometrium was 12.3 × 8 cm^2^ (Figure 1B). However, we found no evidence of endometrial invasion. Chest X-ray revealed no apparent abnormalities, and there was no metastasis to other organs. According to the Cancer Committee of the International Federation of Gynecologists and Obstetricians (FIGO, 2000) (6) GTN staging and classification, the lesion was limited to the uterus, with no distant metastasis. Therefore, we classified the invasive mole as stage I (Table 1), with a prognostic factor of 7. Chemotherapy was prescribed.

**Table 1:** WHO scoring system based on prognostic factors.

**WHO risk factor scoring with FIGO staging**	**0**	**1**	**2**	**4**
Age	<40	>40	—	—
Antecedent pregnancy	Mole	Abortion	Term	
Interval from index pregnancy, months	<4	4–6	7–12	>12
Pretreatment HCG mIU/ml	<10^3^	>10^3^-10^4^	>10^4^-10^5^	>10^5^
Largest tumor size including uterus, cm	—	3–4	≥5	—
Size of metastases including uterus	Lung	Spleen, kidney	Gastrointestinal tract	Brainmliver
Number of metastases identified	—	1–4	5–8	>8
Previous failed chemotherapy	—	—	Single drug	Two or more drugs

*To stage and allot a risk factor score, a patient's diagnosis is allocated to a Stage as represented by a Roman numeral I, II, III, or IV. This is then separated by a colon from the sum of all the actual risk factor scores expressed in Arabic numerals e.g., Stage II:4, Stage IV:9. This Stage and score will be allotted for each patient*.

**Figure 2:**
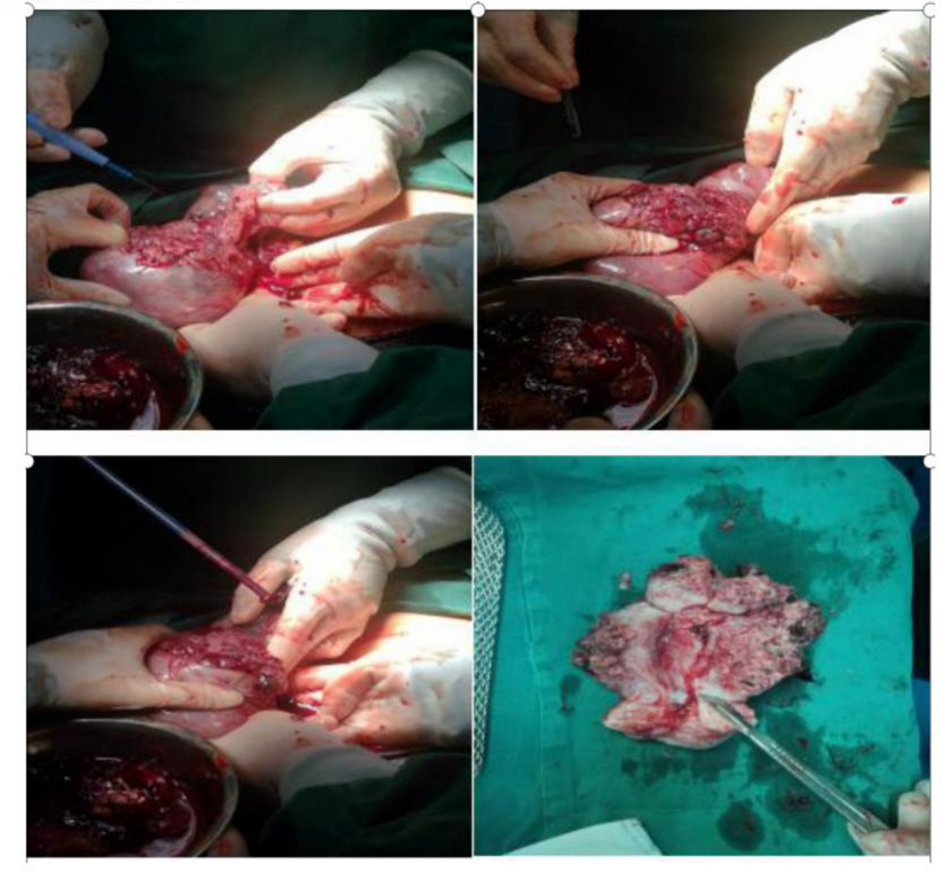
Intraoperatively, the uterus was ruptured, with a breach of about 10 cm, leaving a grape-like tissue. Incision of uterus, the lesion invaded the entire layer of the uterus but not into the cervix and endometrium.

**Figure 3:**
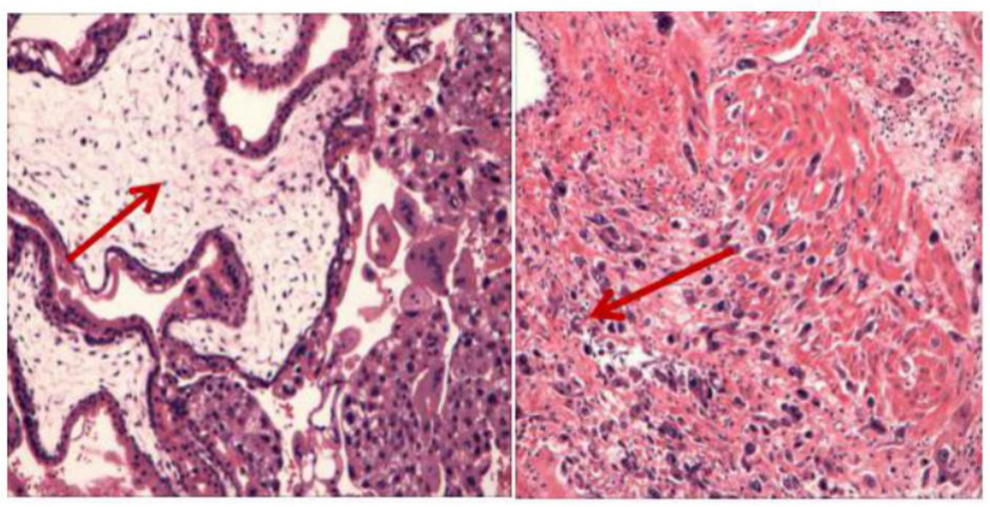
The histopathological examination showed obvious proliferation of trophoblasts and enlarged villi with the formation of a central pool, which invaded the entire posterior wall of the uterus. Coincidence the aggressive mole.

During the preparation of the chemotherapy regimen, the patient had severe pain in the lower abdomen, which spread throughout the stomach, and blood pressure dropped to 80/50 mmHg, heart rate (HR) 63 times/min. Physical examination revealed that all her abdominal muscles were tense and tender, and this was accompanied by rebound tenderness, albeit with no vaginal bleeding. We suspected uterine rupture and immediately subjected the patient to surgical treatment. Intraoperative exploration revealed that the anterior uterine wall had ruptured about 8 cm, and there was evidence of botryoid tissue on the surface. Estimated blood loss was 2,600 ml (Figure 2). Uterine removal was required. Postoperative histopathological results revealed an invasive mole (Figure 3). Subsequently, the patient underwent six cycles of chemotherapy, and follow-up results after chemotherapy were normal.

## Discussion

All invasive moles originate from a hydatidiform mole, and most of them occur within a half year following mole removal. During this period, patients may experience irregular vaginal bleeding and exhibit high HCG levels in their serum. Approximately 15% of all invasive moles metastasize to the lungs or vagina (7). Chemotherapy is the mainstay of invasive mole treatment, with surgery, and radiotherapy is used as supplementary. The cure rate of low-risk GTN can reach 100%, while the cure rate of high-risk GTN can 94% (8). Among them, surgery is used for adjuvant treatment (9). Hysterectomy is an alternative surgical regimen for high risk of GTN patients who do not need to fertile. However, systemic chemotherapy, rather than surgery, is the typical first-choice strategy for treating lung metastases (10).

In this case, the patient still experienced irregular vaginal bleeding 53 days after the abortion. Her serum HCG was higher than 10,000 mIU/ml, and images from b-ultrasound and MR examination revealed a mass that invaded the myometrium, but with no distant metastasis. According to the clinical features, the patient was diagnosed as GTN. High-risk GTN, which is defined as stages II–III (FIGO, 2000) (6) of disease progression with a prognostic score of seven, or FIGO stage IV disease (11), is mainly treated with chemotherapy. Etoposide, methotrexate, actinomycin-D, cyclophosphamide, vincristine (EMA-CO) is the most commonly used initial regimen for treatment of invasive mole or choriocarcinoma (11). However, b-ultrasound and MR examination images revealed a huge lesion in the myometrium, which could not be completely eliminated by chemotherapy alone. Notably, the lesion was so close to the uterine serosa, and was very likely to penetrate the uterus. Rapid growth and massive bleeding of tumors may result in surgical emergency. Uterine perforation due to myometrium invasion is one of these emergent conditions (12). If a patient does not wish fertility, she can have hysterectomy combined with chemotherapy. The patient in this case had no children, so she wanted to be able to sustain a pregnancy and bear a child. A key conundrum in such a case is how to choose an ideal treatment plan to avoid uterine perforation and complete remission. Unfortunately, while we were discussing the treatment plan, the patient developed uterine rupture and was shocked. The uterus cannot be repaired during the operation, and could only be excised.

However, uterine lesion resection combined with subsequent chemotherapy for young women with fertility requirements may be a feasible and safe therapeutic strategy (12), especially young women with fertility requirements and invasive tumors in the myometrium. Lee et al. (13) reported a case of an invasive mole, where a patient was injected with 50 mg methotrexate around 1 month, and her serum HCG levels decreased from 17,008 to 68 mIU/ml. They removed the lesion (about 3.6 × 2.9 × 2.4 cm) by laparoscopy, and finally, 1 year later, the patient was performed at 36 weeks without uterine rupture. Another patient with chemotherapy-resistant GTN was treated with uterine segmental resection followed by hysteroplasty. She had two successful pregnancies after the treatment (13). In placental site trophoblastic tumor (PSTT) research, the Chiofalo et al. (14), reported some cases, though resection lesion combined with successful chemotherapy pregnancies. Some studies consider that lesion resection may increase the risk of tumor metastasis and recurrence, which needs to be carefully evaluated (12).

Through this case, we believe resection of lesion is feasible in management of GTN. In this case, the lesion is too large in the myometrium and invaded the serosa of the uterus. When to choose lesion resection and whether to remove a lesion is the most serious difficulty. Even if chemotherapy is performed first, uterine rupture is likely to occur during chemotherapy. If surgery is performed, the lesion may not be removed entirely. Again, the case was systematically reviewed. The patient had fertility requirements and no distant metastasis. We should try to remove the lesion, and the n chemotherapy after operation. If there are still lesions that cannot be eliminated by chemotherapy, second lesion resection can be considered. In the treatment of GTN, we should consider individualized treatment.

For this case, we recommended the following: (1) After abortion, specimens for pathological examination should be collected, and serum hC G levels monitored until normal and (2) we should be more involved in chemotherapy-assisted surgical therapy for this patient with fertility requirements if the lesion invaded the myometrium.

## Data Availability Statement

The original contributions presented in the study are included in the article/supplementary materials, further inquiries can be directed to the corresponding author.

## Ethics Statement

Written informed consent was obtained from the individual(s), and minor(s)' legal guardian/next of kin, for the publication of any potentially identifiable images or data included in this article.

## Author Contributions

All authors listed have made a substantial, direct, and intellectual contribution to the work and approved it for publication.

## Conflict of Interest

The authors declare that the research was conducted in the absence of any commercial or financial relationships that could be construed as a potential conflict of interest.

## Publisher's Note

All claims expressed in this article are solely those of the authors and do not necessarily represent those of their affiliated organizations, or those of the publisher, the editors and the reviewers. Any product that may be evaluated in this article, or claim that may be made by its manufacturer, is not guaranteed or endorsed by the publisher.
